# Repetitio est mater studiorum – Implementierung von HNO-Fällen in fallbasiertes E-Learning

**DOI:** 10.1007/s00106-023-01409-4

**Published:** 2024-01-05

**Authors:** Jonas Engert, Joy Backhaus, Linda Henig, Bjoern Spahn, Johannes Voelker, Pascal Ickrath, Agmal Scherzad, Kristen Rak, Rudolf Hagen, Sarah König, Stephan Hackenberg, Stefan Kaulitz

**Affiliations:** 1 Klinik und Poliklinik für Hals‑, Nasen- und Ohrenkrankheiten, plastische und ästhetische Operationen, Universitätsklinikum Würzburg, Josef-Schneider-Straße 11, 97080 Würzburg, Deutschland; 2 Institut für Medizinische Lehre und Ausbildungsforschung, Universitätsklinikum Würzburg, Würzburg, Deutschland

**Keywords:** Lehre, Medizinstudierende, Problembasiertes Lernen, Simulationstraining, HNO-Heilkunde, Teaching, Medical students, Problem-based learning, Simulation training, Otorhinolaryngology

## Abstract

**Hintergrund:**

Die deutsche universitäre HNO-Heilkunde hat Bedarf an digitalen Lehrinhalten. Fallbasiertes E‑Learning stellt eine digitale Lehrmethodik dar. Die Datenlage zur studentischen Nutzung von fallbasiertem E‑Learning in der Hochschullehre der HNO-Heilkunde ist limitiert.

**Fragestellung:**

Ziel dieser Arbeit war es festzustellen, in welchem Umfang freiwilliges fallbasiertes E‑Learning von Studierenden der HNO-Heilkunde genutzt wird und welchen Einfluss die Qualität des E‑Learnings auf die Motivation zur E‑Learning-Nutzung und auf das Interesse für die HNO-Heilkunde hat.

**Material und Methoden:**

Es wurden 15 freiwillige E‑Learning-Fälle erstellt, welche auf den Inhalten der HNO-Vorlesung im Wintersemester 2022/2023 basierten. Im Anschluss erfolgte eine deskriptive Auswertung der Nutzungsstatistiken der Fälle von 157 Studierenden. Ebenso wurde eine Evaluation der Qualität des E‑Learnings sowie der Motivation zur Durchführung und des Interesses für die HNO-Heilkunde mittels eines freiwilligen Fragebogens durchgeführt.

**Ergebnisse:**

Freiwilliges fallbasiertes E‑Learning wurde von 66 % der Studierenden in unterschiedlichem Ausmaß genutzt. Die Qualität des E‑Learnings korrelierte signifikant mit der Motivation zur Durchführung und dem Interesse für die HNO-Heilkunde.

**Schlussfolgerung:**

Lehrinhalte der HNO-Heilkunde lassen sich suffizient in fallbasiertes E‑Learning implementieren und zeichnen sich durch eine zufriedenstellende studentische Akzeptanz aus. Die Eingliederung sollte auf eine qualitativ hochwertige Art und Weise erfolgen, um die Motivation zur Durchführung und das Interesse an der HNO-Heilkunde zur erhöhen.

**Zusatzmaterial online:**

Die Online-Version dieses Beitrags (10.1007/s00106-023-01409-4) enthält den Fragebogen zur Evaluation der HNO-CaseTrains.

## Abstract (en)

**Background:**

German university otorhinolaryngology has a need for digital teaching content. Case-based e‑learning represents a digital teaching methodology. The data on student use of case-based e‑learning in university teaching of ENT medicine are limited.

**Objective:**

The aim of this work was to determine the extent to which voluntary case-based e‑learning is used by otolaryngology students and what influence the quality of the e‑learning has on motivation to use e‑learning and on the interest in otolaryngology.

**Materials and methods:**

Fifteen voluntary e‑learning cases were created based on the content of the ENT lecture in the winter semester 2022/2023. Subsequently, a descriptive evaluation of the usage statistics of the cases of 157 students was conducted. Likewise, an evaluation of the quality of the e‑learning as well as the motivation to complete it and the interest in otorhinolaryngology was carried out using a voluntary questionnaire.

**Results:**

Voluntary case-based e‑learning was used to varying degrees by 66% of the students. The quality of e‑learning correlated significantly with the motivation and the interest in otolaryngology.

**Conclusion:**

The teaching content of otorhinolaryngology can be implemented sufficiently in case-based e‑learning and is characterized by satisfactory student acceptance. Integration should be accomplished in a high-quality manner to increase motivation and interest in otorhinolaryngology.

## Digitale Lehrkonzepte

Unter E‑Learning werden alle Lernformen verstanden, bei welchen digitale Medien zur Wissensvermittlung angewendet werden [1]. Hingegen beschreibt Blended-Learning die didaktisch sinnhafte Verknüpfung von digitaler und Präsenzlehre [2]. Der Bedarf an E‑Learning und Blended-Learning in der universitären Ausbildung der HNO-Heilkunde in Deutschland ist unbestritten [3]. Eine nationale Umfrage aus dem Jahr 2015 ergab, dass digitale Lernprogramme, die komplexe klinische Patientenfälle abbilden, nur in geringem Prozentsatz zum Einsatz kamen [3]. Die SARS-CoV-2-Pandemie wird als Katalysator der Digitalisierung in der medizinischen Lehre gewertet und führte zu einer zügigen Implementation von digitalen Lehrkonzepten [4]. Dieser Trend wurde in einer Umfrage der Direktoren der deutschen Universitäts-HNO-Kliniken bestätigt [5]. Offergeld et al. konnten bei deutschen Studierenden eine positive Einstellung gegenüber Aspekten der Digitalisierung im Bereich der HNO-Heilkunde feststellen [6]. In den vergangenen Jahren wurden verschiedene digitale Lehrkonzepte entwickelt, die den Entwicklungsprozess von reiner Präsenzlehre hin zu hybriden Umsetzungsformen skizzieren und digitale Lernaktivitäten gewinnbringend einbeziehen [7]. Beispielsweise wurde eine Lernplattform entwickelt, die umfänglich die curriculare Lehre der HNO-Heilkunde online abbildet [8]. Ebenso wurde videobasierter Distanzunterricht entwickelt, um den pandemiebedingten Kontaktbeschränkungen gerecht zu werden. Diese Unterrichtsform wurde von den Studierenden positiv aufgenommen und stellt im Bereich der HNO-Heilkunde eine gute Alternative zu Präsenzveranstaltungen dar [9]. Bemerkenswert war die hohe Akzeptanz virtueller Studieninhalte und Umsetzungsformen, die von den Studierenden sogar teilweise besser als der konventionelle Präsenzunterricht beurteilt wurden [10]. Außerdem konnten Verse et al. zeigen, dass bei der Analyse der Klausurergebnisse kein qualitativer Unterschied zwischen digitaler und analoger Lehre vorlag [11]. Theoretisches Basiswissen der HNO-Heilkunde lässt sich digital, im Vergleich zu praktischen Fähigkeiten, besser vermitteln [11].

Fallbasiertes E‑Learning ist eine zunehmend genutzte Option [12, 13]. Auch in der HNO-Heilkunde wurden entsprechende Formate etabliert [8, 14]. Es handelt sich um eine medizindidaktische Methode, die bereits 1912 von James Lorrain Smith in der Pathologie entwickelt wurde und sich als lernwirksam in der Ausbildung von Gesundheitsberufen bewährt hat [15, 16]. Im Verlauf der Jahre erfolgte eine zunehmende Digitalisierung, welche sich mindestens genauso effektiv wie fallbasierte Präsenzlehre zeigte [17, 18]. In dem sich entwickelnden Feld des fallbasierten E‑Learnings ist es daher von besonderem Interesse, das Nutzungsverhalten in Kombination zu bestehenden Lehrveranstaltungen sowie die Lernvorlieben der Studierenden zu analysieren [19].

Das Ziel der vorliegenden Untersuchung bestand darin, HNO-Fälle für ein bestehendes E‑Learning-System zu entwickeln und darüber die systematische Wissensvermittlung der Präsenzlehre mit praxisrelevanten und flexibel umsetzbaren Lernaktivitäten zu ergänzen. Dabei sollten das Nutzungsverhalten des freiwilligen Angebots und das Leistungsergebnis der Studierenden analysiert werden. Ferner standen im Rahmen der Evaluation die Bewertung der E‑Learning-Fälle und das Lernerleben im Fokus.

## Material und Methoden

### CaseTrain-Erstellung

**Figure 1:**
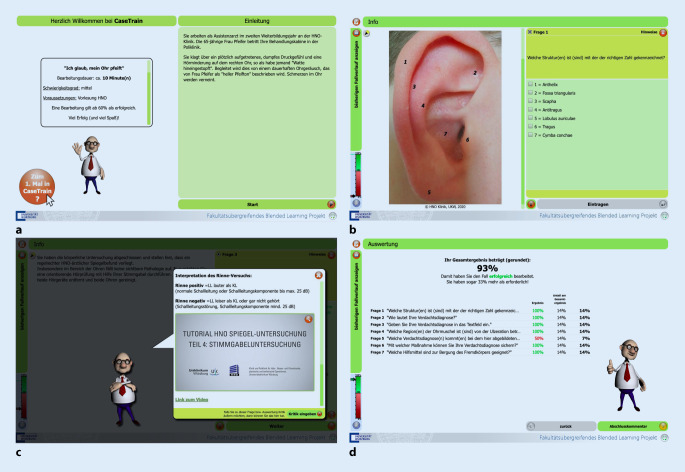

CaseTrain wurde an der Julius-Maximilians-Universität Würzburg entwickelt, um die Implementierung von fallbasiertem Lernen in Blended-Learning-Konzepten zu ermöglichen [20]. Es handelt es sich um einen webbasierten Fallplayer, der in die Lehrangebote der Plattform WueCampus (https://wuecampus2.uni-wuerzburg.de) integriert ist. Die Programmierung umfasst eine Shockwave-Flash-Datei, die mithilfe des Adobe® Flash Player Plugin (Adobe Inc., San José, CA, USA) wiedergegeben wird. Die CaseTrain-Fälle (CaseTrains) wurden nach einem einheitlichen Schema konzeptioniert: Die Einleitung führte in den klinischen Fall ein (Abb. 1a), daran schlossen sich zur Selbsttestung Fragen (6–12) mit Einbezug von Bild- und Videomaterial an (Abb. 1b). Nach jeder Beantwortung gab es eine Rückmeldung mit Erläuterungen zu den korrekt und inkorrekt ausgewählten Optionen (Abb. 1c). CaseTrains konnten beliebig häufig bearbeitet und neugestartet werden. Ab 60 % korrekt ausgewählter Antworten galt der jeweilige Fall als erfolgreich abgeschlossen (Abb. 1d). Es wurden Einfach‑, Mehrfach- und Textfragen zur Testung genutzt. Es wurden 15 HNO-Patientenfälle implementiert, die sich nach den Vorlesungsthemen richteten. Bei der Erstellung der Fälle wurde darauf geachtet, dass ausschließlich klausurrelevante Themen dargestellt wurden.

### Studierende, Setting und Datenerhebung

Im Wintersemester 2022/2023 wurden die CaseTrains als freiwilliges Zusatzangebot allen 157 Medizinstudierenden des 7. Semesters über die Lernplattform WueCampus (https://wuecampus2.uni-wuerzburg.de) zugänglich gemacht. Im Rahmen der Einführungsveranstaltung zu Semesterbeginn wurden die Studierenden auf das Zusatzangebot hingewiesen. Die Nutzungsstatistik wurde über die Programmfunktion „CaseTrain-Manager“ erfasst. Die Fallgebenden hatten die Möglichkeit, die Gesamtanzahl der abgeschlossenen Fallbearbeitungen und die Anzahl der erfolgreich und erfolglos abgeschlossenen Bearbeitungen zu erfassen. Zudem wurde die Bearbeitungszeit und das prozentuale Leistungsergebnis der abgeschlossenen Patientenfälle erhoben.

### Evaluation

Die Studierenden wurden am Semesterende mit dem Online-Evaluationsinstrument EvaSys® (evasys GmbH, Lüneburg, Deutschland) befragt. Der Fragebogen umfasste 4 Abschnitte:

1. Demografie (Alter und Geschlecht),
2. Qualität der CaseTrains und Präferenzen zum E‑Learning,
3. Interesse am Fach HNO-Heilkunde und Motivation zur Fallbearbeitung und
4. Freitextkommentare zu positiven Aspekten und Verbesserungsvorschlägen.

Bei den geschlossenen Items kam eine Likert-Skala mit den Polen 1 (trifft überhaupt nicht zu) bis 5 (trifft voll zu) zur Anwendung. Der Fragebogen ist im Online-Zusatzmaterial dargestellt.

### Statistische Auswertung

Die deskriptive Statistik umfasste den Mittelwert (M) und die Standardabweichung (S). Die Testgüte wurde mittels Cronbach-Alpha (α), Trennschärfe (r_it_) und Schwierigkeit (P) bestimmt. Werte von α > 0,70; r_it_ > 0,30 und P von 0,20–0,80 galten als hinreichend für inferenzstatistische Berechnungen [21]. Zur Darstellung der Korrelation wurde das Pearson‑r verwendet. Die Kommentare der Studierenden wurden qualitativ zusammengefasst. Die Auswertungen und Grafiken wurden mit Microsoft® Excel 2023 V16.70 (Fa. Microsoft Corporation, Redmond/WA, USA) und R Statistical Software (v3.6.1; R Core Team) erstellt.

## Ergebnisse

### Auswertung der Nutzungsstatistiken

**Figure 2:**
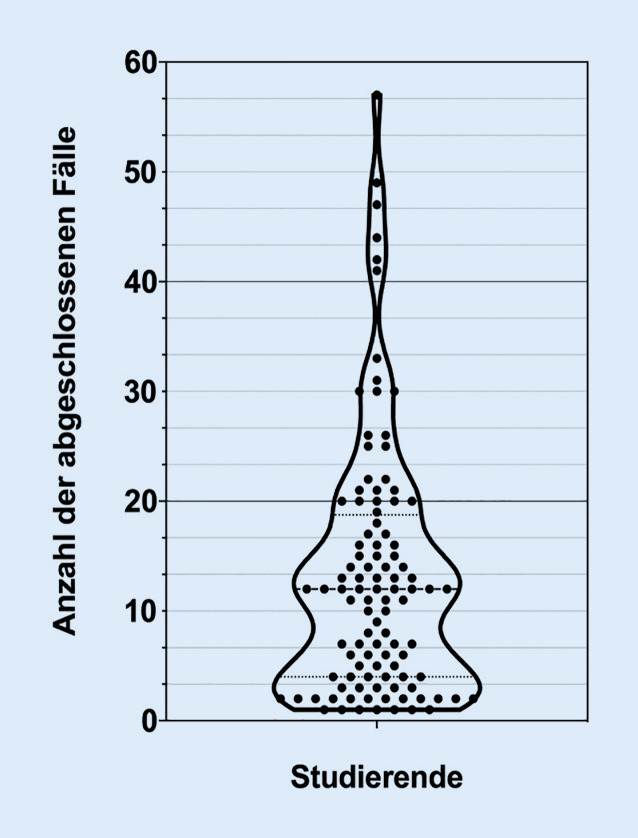

Die freiwillige Nutzung wurde von 104 Studierenden (66,24 %) durchgeführt. Insgesamt wurden 1369 Fälle abgeschlossen. Dabei wurden 773 dieser Fälle erfolgreich (56 %) und 596 Fälle erfolglos (44 %) abgeschlossen. Pro das System nutzender Person wurden durchschnittlich 13,16 Fälle abgeschlossen. Die niedrigste Bearbeitungsanzahl lag bei einem abgeschlossenen CaseTrain und die höchste bei 57 abgeschlossenen CaseTrains (Abb. 2). Von den nutzenden Studierenden wiesen 24,03 % mindestens 20 oder mehr und 10,57 % mindestens 30 oder mehr abgeschlossene Fälle auf.

**Figure 3:**
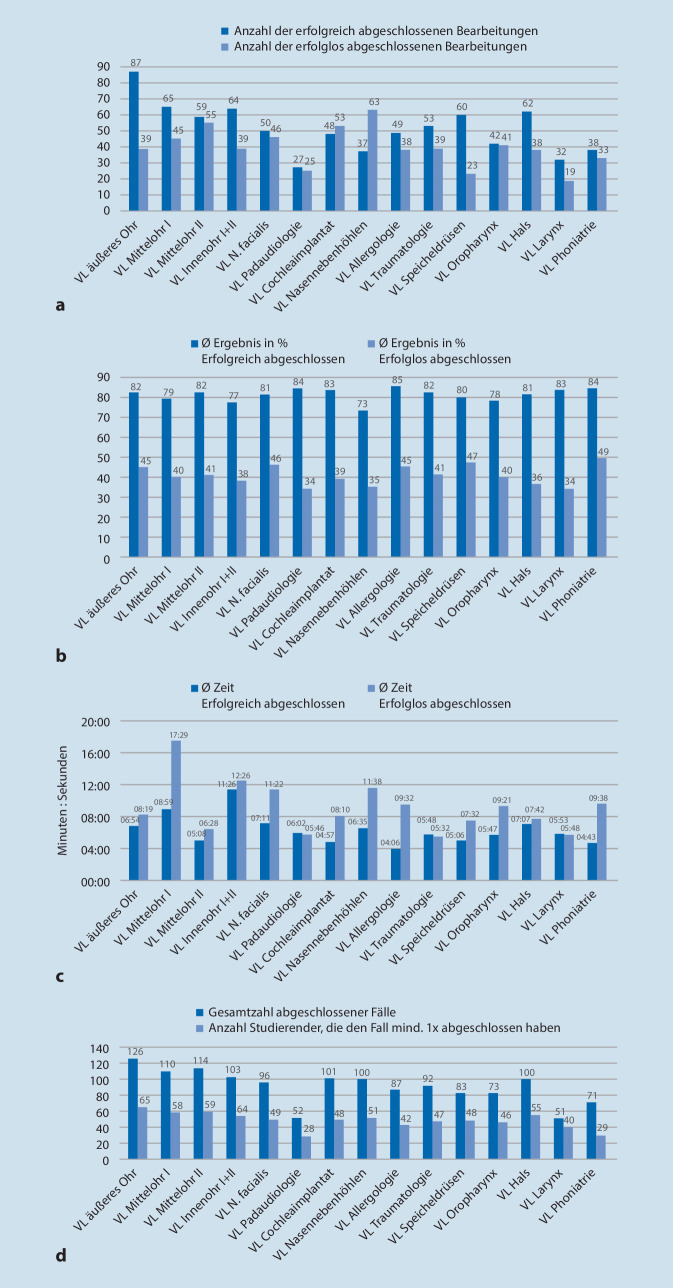

Es zeigte sich, dass fast ausschließlich mehr erfolgreiche als erfolglose Fälle pro Thema zum Abschluss kamen (Abb. 3a). Die prozentuale Auswertung der Ergebnisse erbrachte eine gleichbleibende Leistung der Studierenden im Semesterverlauf (Abb. 3b). Die durchschnittliche Leistung der Studierenden bei erfolgreich abgeschlossenen Fällen betrug 80,93 und 40,66 % bei erfolglosem Abschluss. Erfolgreich abgeschlossene Fälle (durchschnittlich 6 min, 22 s) wurden kürzer bearbeitet als erfolglos abgeschlossene Fälle (durchschnittlich 9 min, 7 s; Abb. 3c). Es zeigte sich tendenziell eine abnehmende Nutzungshäufigkeit im Verlauf des Semesters (Abb. 3d, chronologische Anordnung der Vorlesungsthemen). In nahezu allen Fällen war eine mehrfache Bearbeitung der Fälle erkennbar (durchschnittlich 2,16-fach), da die Gesamtanzahl abgeschlossener Fälle (durchschnittlich 91,26) deutlich höher ausfiel als die Anzahl der Studierenden, die den Fall mindestens einmal abgeschlossen (durchschnittlich 47,93) hatten (Abb. 3d).

### Auswertung der Evaluationsbögen

An der Befragung nahmen insgesamt 40 Studierende (25,47 %) teil, davon waren 24 Frauen und 16 Männer. Der Altersmedian lag bei 24,5 Jahren. Die Ergebnisse der Testgütekriterien zum Abschnitt „Qualität der CaseTrains und Präferenzen zum E‑Learning“ sind in Tab. 1 dargestellt. Der α‑Wert fiel mit 0,80 für die Skala „Qualität der CaseTrains und Präferenzen zum E‑Learning“ wünschenswert aus (Tab. 1).

**Table 1:** 

Qualität der CaseTrains und Präferenzen zum E‑Learning (i = 6)	M	S	P	r_it_	α falls entfernt
Verständlichkeit Fragen	4,49	0,64	0,90	0,38	0,80
Verständlichkeit Erläuterungen	4,50	0,65	0,90	0,37	0,80
Hilfe für Klausurvorbereitung	4,00	1,01	0,80	0,83	0,69
Thema aus Vorlesung bekannt	3,50	0,82	0,70	0,49	0,78
Bevorzugen von E‑Learning gegenüber Präsenzlehre	3,00	1,06	0,60	0,60	0,76
Vermehrte Anwendung von CaseTrains erwünscht	4,08	1,25	0,81	0,70	0,73

Durchschnittliche Inter-Item-Korrelation = 0,39; gesamtes Cronbach-α = 0,80

Es erfolgte die Auswertung der durch eine Likert-Skala (1 = „trifft überhaupt nicht zu“ bis 5 = „trifft vollständig zu“) erhobenen Daten

*α falls entfernt* Cronbach‑α,* M* Mittelwert, *P* Schwierigkeit, *r*_*it*_ Trennschärfe, *S* Standarddeviation

Die Ergebnisse der Testgütekriterien zum Abschnitt „Motivation zur Fallbearbeitung“ sind in Tab. 2 dargestellt. Die Bewertung der Testgütekriterien der Skala „Motivation zur Fallbearbeitung“ fiel mit einem α‑Wert von 0,84 wünschenswert aus. Die Analysen des Items „Interesse am Fach“ erbrachten einen Mittelwert von 3,08 (S 1,12; P 0,62).

**Table 2:** 

Motivation zur Fallbearbeitung (i = 2)	M	S	P	r_it_	α falls entfernt
Motivation für CaseTrains vor dem Semester (retrospektiv)	2,92	0,94	0,58	NV	NV
Motivation für CaseTrains nach dem Semester	3,48	1,09	0,70	NV	NV

Inter-Item-Korrelation = 0,73; gesamtes Cronbach-α = 0,84

Es erfolgte die Auswertung der durch eine Likert-Skala (1 = „trifft überhaupt nicht zu“ bis 5 = „trifft vollständig zu“) erhobenen Daten

*α falls entfernt* Cronbach‑α,* M* Mittelwert, *NV* nicht vorhanden,* P* Schwierigkeit, *r*_*it*_ Trennschärfe, *S* Standarddeviation

**Figure 4:**
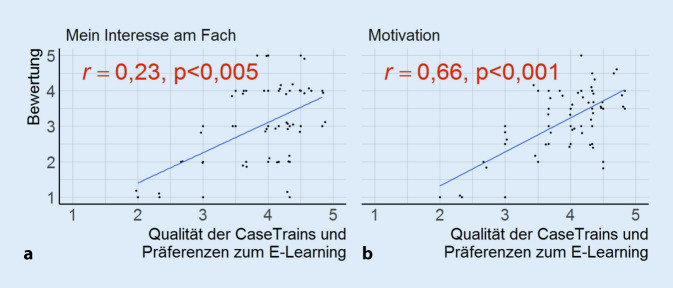

Das Item „Interesse am Fach“ und die beiden Items der „Motivation zur Fallbearbeitung“ korrelierten jeweils positiv mit „Qualität der CaseTrains und Präferenzen zum E‑Learning“ (Abb. 4).

## Diskussion

### Gezielte Nutzung

Es zeigte sich eine zufriedenstellende, aber ausbaufähige Nutzung trotz der Freiwilligkeit der CaseTrains (66,24 %). Fischer et al. Verglichen an deutschen Hochschulen unterschiedliche Strategien zur Implementierung von E‑Learning in das medizinische Curriculum [22]. Hierbei zeigte sich eine sehr niedrige Nutzung (8,8 %) bei Freiwilligkeit ohne Relevanz zum Examen oder der Lehrveranstaltung. Hingegen zeigte sich bei Freiwilligkeit mit Examensrelevanz eine sehr hohe Nutzung (90,7 %). Die geringere prozentuale Nutzung in dieser Studie trotz gleicher Voraussetzungen (Freiwilligkeit und Prüfungsrelevanz) bietet Raum für Verbesserungsansätze. Eine Einschränkung ist die nur einmalige Bewerbung der CaseTrains in der Einführungsvorlesung. Wiederholender Bezug auf die CaseTrains und deren Lehrinhalte im Rahmen der Präsenzveranstaltungen könnte die Nutzungshäufigkeit steigern [22]. Die CaseTrains wurden durchschnittlich als sehr hilfreich zur Klausurvorbereitung bewertet (Tab. 1). Fallbasiertes E‑Learning wird v. a. dann genutzt, wenn es auf freiwilliger Basis und mit thematischer Relevanz zum Leistungsnachweis etabliert wird [12]. Bei vergleichbaren Projekten aus dem Kopf-Hals-Bereich stellte sich heraus, dass die Relevanz für die Klausur entscheidend für die Teilnahme an digitalen Lehrangeboten ist [14]. Die Feststellung, dass Studierende ihren Lernfokus dem Anforderungsspektrum anpassen, ist eine bedeutende Beobachtung, die bei der Erstellung der Curricula sowie der Leistungsnachweise bedacht werden muss, und unterstreicht die Aussage „assessment drives learning“ von Wass et al. [23]. Diese Aussage beschreibt, dass die Bewertung und Prüfung des Lernens den am besten geeigneten Motor bei der Gestaltung des Lehrplans darstellt [23]. Plakativ ausgedrückt, betont die Aussage „assessment drives learning“, dass Studierende v. a. die Lehrinhalte lernen, über welche sie geprüft werden [23]. Insgesamt nutzte eine Subgruppe E‑Learning also gezielt und häufig bei zufriedenstellender Gesamtnutzung.

Außerdem zeigte sich bei der Auswertung der CaseTrain-Statistiken eine kürzere Bearbeitungszeit von erfolgreich bearbeiteten Fällen (Abb. 3c) und eine höhere Anzahl an abgeschlossenen Bearbeitungen bei jedem Thema als die Anzahl an Studierenden, die den Fall mindestens einmal bearbeitet haben (Abb. 3d). Ebenso hatten 10,57 % der Nutzenden 30 oder mehr Fälle abgeschlossen (Abb. 2). Dies weist auf einen hohen Anteil an Mehrfachbearbeitungen einer relevanten Subgruppe hin. Es ist bekannt, dass eine Subgruppe an Studierenden gezielt Online-Kurse nutzt, um Lehrinhalte zu rekapitulieren im Sinne des Spiralcurriculums [24]. Dieses stellt ein didaktische Lehrkonzept dar und beschreibt die mehrfache Wiederholung des Lehrinhalts mit steigendem Lernniveau im zeitlichen Verlauf. Der Lehrinhalt wird somit nicht linear angeordnet, sondern wiederholend und zunehmend differenziert gelehrt [25]. Ferner wurde ein Anteil an Studierenden beschrieben (als „Holisten“ bezeichnet), die signifikant dazu neigen, Online-Lehrinhalte mehrfach zu wiederholen [26]. Diese Interpretation wird von den qualitativen Antworten der Studierenden auf die offenen Fragen gestützt: „Ich finde es gut, zur Selbstkontrolle Fragen zum Lernstoff bearbeiten zu können“. Ein weiterer Trend, der sich in der Auswertung andeutet, ist eine abnehmende Nutzung der CaseTrains im Verlauf des Semesters (Abb. 3d). Dies lässt vermuten, dass Studierende, die keinen Nutzen von den CaseTrains erfahren, das Angebot im Verlauf des Semesters weniger nutzen.

### Qualitätsabhängiges Interesse

Die Rücklaufquote der freiwilligen Evaluationsbogen war vergleichbar mit ähnlichen Studien [10, 27]. Die Geschlechts- und Altersverteilung war sehr gut mit der Kohorte von Medizinstudierenden in Deutschland im 7. Semester vereinbar [28]. Der Wunsch nach vermehrter Anwendung von E‑Learning im HNO-Bereich ist bekannt (Tab. 1; [14]).

Die Studierenden nahmen durchschnittlich die Fragen und Erläuterungen der CaseTrains als sehr strukturiert wahr (Tab. 1). Die Ergebnisse der Korrelationsanalyse weisen darauf hin, dass eine strukturierte Erstellung und Einbindung der Lehrinhalte in die CaseTrains einen bedeutenden Effekt auf sowohl die Motivation zur Durchführung der CaseTrains als auch auf das Interesse an der HNO-Heilkunde hat (Abb. 4). Lang et al. evaluierten das digitale Lehrprogramm der Universitäts-HNO-Klinik Freiburg, welches Vorlesungen, Seminare, Praktika und Übungsfragen beinhaltet, und stellten fest, dass die Lehrmodule in hohem Maße genutzt wurden und das Lehrprogramm lernmotivationsfördernd sowie interessesteigernd wirkte [8]. Die Qualität des E‑Learnings steuert also Motivation und Interesse.

CaseTrains ermöglichen den Studierenden, zu jedem beliebigen Zeitpunkt ihren Lernfortschritt zu überprüfen und bei Bedarf ein strukturiertes Feedback auf die Antwortmöglichkeiten einzusehen. Dies reduziert die Komplexität des Lernens und wirkt sich positiv auf die weitere Anwendung von E‑Learning aus [29]. Die qualitativen Aussagen der Studierenden unterstreichen die Bedeutung des Inhalts und der Struktur: „Falldarstellung passend zu Vorlesungsthemen, mit Bildern und verständlichen Erklärung und Verlauf der Behandlung“.

## Fazit für die Praxis

- Die Implementierung von HNO-Lehrinhalten in fallbasiertes E‑Learning ist sehr gut realisierbar und zeichnet sich durch eine zufriedenstellende studentische Bearbeitungsquote aus.
- Eine relevante Gruppe der Studierenden nutzt fallbasiertes E‑Learning mehrfach zum Rekapitulieren der Lehrinhalte im Verlauf des Semesters.
- Je besser fallbasiertes E‑Learning in der Qualität wahrgenommen wird, desto höher ist die Motivation der Studierenden zur Bearbeitung der Fälle.
- Auch das empfundene Interesse am Fach HNO-Heilkunde ist mit den hohen Bewertungen in der Evaluation vergesellschaftet.

### Supplementary Information




