# Supplementary material for: Repetitio est mater studiorum—implementation of ENT cases in case-based e-learning
Source: HNO. 2024 Jan 5;72(3):166–72. [Article in German] doi: 10.1007/s00106-023-01409-4 (PMC10879404; doi:10.1007/s00106-023-01409-4)
Supplement: Supplementary file 1 [file 106_2023_1409_MOESM1_ESM.pdf]

MUSTER

EvaSys	Evaluation der HNO CaseTrain	Electric Paper

Bitte so markieren: ☐ ☒ ☐ ☐ ☐ Bitte verwenden Sie einen Kugelschreiber oder nicht zu starken Filzstift. Dieser Fragebogen wird maschinell erfasst.

Korrektur: ☐ ■ ☐ ☒ ☐ Bitte beachten Sie im Interesse einer optimalen Datenerfassung die links gegebenen Hinweise beim Ausfüllen.

1. Demographie

1.1 Wie alt sind Sie?

1.2 Welches Geschlecht haben Sie? ☐ männlich ☐ weiblich ☐ divers

2. Inhalt und Struktur

2.1	Die Fragestellungen im den CaseTrains waren verständlich formuliert.	Trifft überhaupt nicht zu. ☐	☐	☐	☐	☐	Trifft voll zu.
2.2	Die Erläuterungen zu den Antwortmöglichkeiten der CaseTrains waren verständlich formuliert.	Trifft überhaupt nicht zu. ☐	☐	☐	☐	☐	Trifft voll zu.
2.3	Die CaseTrains waren ein hilfreiches Element zur Vorbereitung für die HNO-Klausur.	Trifft überhaupt nicht zu. ☐	☐	☐	☐	☐	Trifft voll zu.
2.4	Der thematische Inhalt der CaseTrains war mir aus den Vorlesungen bekannt.	Trifft überhaupt nicht zu. ☐	☐	☐	☐	☐	Trifft voll zu.
2.5	Durch die CaseTrains wurde mir Wissen vermittelt, welches mir aus den Vorlesungen nicht bekannt war.	Trifft überhaupt nicht zu. ☐	☐	☐	☐	☐	Trifft voll zu.
2.6	Ich bevorzuge fallbasiertes E-Learning (CaseTrain) im Vergleich zu fallbasierter Präsenzlehre (bspw. Seminar mit Fallbesprechungen).	Trifft überhaupt nicht zu. ☐	☐	☐	☐	☐	Trifft voll zu.
2.7	CaseTrains sollten vermehrt Anwendung finden, um Lehrinhalte aus Vorlesungen zu überprüfen und zu rekapitulieren.	Trifft überhaupt nicht zu. ☐	☐	☐	☐	☐	Trifft voll zu.

3. Motivation

3.1	Mein Interesse für das Fach Hals-, Nasen-, Ohrenheilkunde wurde durch die CaseTrains gesteigert.	Trifft überhaupt nicht zu. ☐	☐	☐	☐	☐	Trifft voll zu.
3.2	Meine Motivation zur Nutzung von CaseTrains **vor** dem Semester war:	Sehr niedrig. ☐	☐	☐	☐	☐	Sehr hoch.
3.3	Meine Motivation zur Nutzung von CaseTrains **nach** dem Semester war:	Sehr niedrig. ☐	☐	☐	☐	☐	Sehr hoch.

4. Lob, Kritik und Feedback

MUSTER

4. Lob, Kritik und Feedback [Fortsetzung]

4.1 Was fandest du besonders gut an den CaseTrains der HNO-Heilkunde?

4.2 Hast du konkrete Verbesserungsvorschläge für die CaseTrains der HNO-Heilkunde?
